# Repeatability of the Vibroarthrogram in the Temporomandibular Joints

**DOI:** 10.3390/s22239542

**Published:** 2022-12-06

**Authors:** Adam Łysiak, Tomasz Marciniak, Dawid Bączkowicz

**Affiliations:** 1 Faculty of Electrical Engineering, Automatic Control and Computer Science, Opole University of Technology, 45-758 Opole, Poland; 2 Department of Rehabilitation, Józef Piłsudski University of Physical Education in Warsaw, 00-809 Warsaw, Poland; 3 Faculty of Physical Education and Physiotherapy, Opole University of Technology, 45-758 Opole, Poland

**Keywords:** vibroarthrography, VAG, joint vibration analysis, JVA, temporomandibular disorders, TMD, TMJ, joint sounds, repeatability, intraclass correlation coefficient

## Abstract

Current research concerning the repeatability of the joint’s sounds examination in the temporomandibular joints (TMJ) is inconclusive; thus, the aim of this study was to investigate the repeatability of the specific features of the vibroarthrogram (VAG) in the TMJ using accelerometers. The joint sounds of both TMJs were measured with VAG accelerometers in two groups, study and control, each consisting of 47 participants (*n* = 94). Two VAG recording sessions consisted of 10 jaw open/close cycles guided by a metronome. The intraclass correlation coefficient (ICC) was calculated for seven VAG signal features. Additionally, a k-nearest-neighbors (KNN) classifier was defined and compared with a state-of-the-art method (joint vibration analysis (JVA) decision tree). ICC indicated excellent (for the integral below 300 Hz feature), good (total integral, integral above 300 Hz, and median frequency features), moderate (integral below to integral above 300 Hz ratio feature) and poor (peak amplitude feature) reliability. The accuracy scores for the KNN classifier (up to 0.81) were higher than those for the JVA decision tree (up to 0.60). The results of this study could open up a new field of research focused on the features of the vibroarthrogram in the context of the TMJ, further improving the diagnosing process.

## 1. Introduction

There are two general means of assessing motion in human joints – quantity and quality. The quantity of movement can be measured with a simple ruler or a goniometer, but also with highly specialized motion capture system such as VICON^®^ [1,2,3].

**Figure 1:**
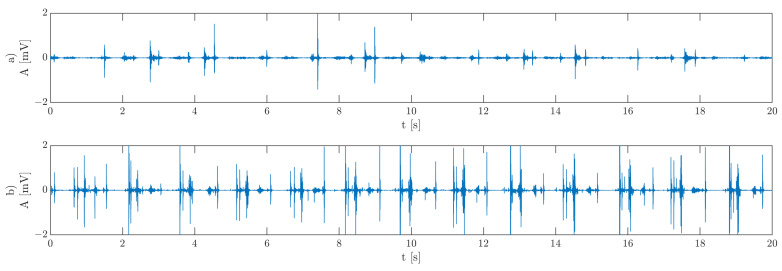
Exemplary VAG signal for (**a**) asymptomatic and (**b**) symptomatic temporomandibular joints.

The quality of the movement is rarely analyzed due to fewer available diagnostic devices, with vibroarthrography (VAG) being one of them [4,5,6]. It is a tool for assessing the quality of motion in human joints [7,8,9]. It has been used for assessing joint’s sounds, for example, in the knees, ankles, and temporomandibular joints (TMJ) [5,10,11]. Figure 1 shows exemplary vibroarthrograms of TMJ joints: Figure 1a, asymptomatic, and Figure 1b, symptomatic.

Temporomandibular disorders (TMD) are the most common cause of orofacial pain [12]. TMD is an umbrella term for different symptoms that may occur in the orofacial area, especially in temporomandibular joints [12]. It is estimated that the prevalence of TMD in the general population is between 3 and 12% [13,14,15], mostly affecting women up to 4–8 times more often than men, of an age between 16 and 45 years [16,17,18,19].

Among the pain of mastication muscles and/or pain of the TMJ itself, joint sounds are one of the most frequent intra-articular joint disorders in this joint with a prevalence between 21.1% and 73.3% [20]. According to the Diagnostic Criteria for Temporomandibular Disorders (DC/TMD), which are the most frequently used diagnostic protocol for TMD for both scientific research and clinical practice, a joint’s sounds may manifest as clicks, crepitus, or eminence clicks [1]. Joint sounds mainly concern younger patients, i.e., below 27 years old [21].

There are two main clinical methods of examining TMJ’s sounds. One of them is a simple tactile palpation also used in the (R)DC/TMD protocols [22,23], while the other is an examination using a stethoscope [24]. Previous research showed that both methods present low ICC values and a high number of false-positive results regarding crepitus [7]. The above-mentioned premise has led to searching for alternative and more objective examination methods regarding joint sounds in the TMJ [25,26,27]. VAG became one of the potential solutions.

Since the palpatory assessment of mechanical vibrations produced by a moving joint is subjective and seems inaccurate in its form [6,28,29,30], the usage of VAG would be an attempt to render the examination more objective.

The most commonly used device for recording and analyzing vibroacoustic signals is a joint vibration analysis (JVA) system, which was designed to examine temporomandibular joints, and consists of two accelerometers and the accompanying software enabling registration and signal analysis [4,5,31,32]. The built-in software determines features characterizing vibroacoustic signals on the basis of the frequency spectrum (features described in the following section). On the basis of these features, the classification of the TMJ pathology in the form of a JVA decision tree was developed [33].

VAG is based on the analysis of vibroacoustic signals produced by the friction created inside the joint. Previous studies regarding knee joints showed that OA knees have a greater frequency, higher peaks, and longer duration concerning the vibroacoustic emissions compared to healthy knees [6,34,35]. An up-to-date VAG is not only used to determine the condition of the cartilage, but also for other intra-articular elements of the joints, such as the menisci in the knee joint and the temporomandibular joint’s disc [5,36]. VAG gives the opportunity to dynamically examine joint function in movement, unlike static imaging showing only the structure. Information obtained by both means could be beneficial for the diagnosing process and treatment options. Additionally, VAG is a very cost-effective tool to incorporate into the process.

Some authors indicated the interpretation subjectivity of vibroacoustic signal analysis, emphasizing the low intrarater reliability [7]. On the other hand, the latest papers showed good and very good repeatability expressed with the intraclass correlation coefficient (ICC) [4,6,28,31,37].

Due to inconclusive research results, the aim of this study was to investigate the repeatability of the specific features of the vibroarthrogram in temporomandibular joints using accelerometers. Additionally, a k-nearest-neighbor classifier was defined to differentiate TMJ VAG signals into symptomatic and asymptomatic groups, and was more accurate than the state of the art.

Currently, only a few features describing the vibration signal of the TMJ joint are defined. By providing evidence for the repeatability of a signal in the measurement chain proposed in this study, a whole new field of research is opened up. In future research, it would be possible to define VAG features that allow for the better classification of a signal (that is, joint diagnosis). VAG signal classification could also be unified with clinical and scientific examination protocols, such as the (R)DC/TMD questionnaire, improving the clinical reasoning process in differentiating pathologies, especially since the JVA decision tree is based on Piper’s classification and is not the most used (R)DC/TMD protocol [38,39]. In addition, the measurement chain described in this publication is used in the diagnosis of other joints, specifically the knee joint, demonstrating the versatility of the described approach. Thus, it is possible to study vibroarthrographic signals in the context of other, as-yet unexplored, joints.

## 2. Materials and Methods

### 2.1. Material

The study comprised 94 subjects divided into two groups on the basis of the presence/absence of joint sounds (clicks representing a disc displacement with reduction according to RDC/TMD) examined by palpation: study group (*n* = 47, 10 men, 37 women; age mean 27.5 (SD 5.6) years)—participants with a presence of joint sounds in the clinical examination, and control group (*n* = 47, 15 men, 32 women; age mean 26.8 (SD 6.8) years)—participants without any sounds or other TMD symptoms in the clinical examination. The female-to-male (FM ) ratio for the examination group was 3.7:1; for the control group, it was 2.1:1. There were no significant differences between the groups regarding age and sex (*p* > 0.05). The group allocation was based on the RDC/TMD examination protocol. The study obtained the approval of the ethical committee of the Józef Piłsudski University of Physical Education, Warsaw, Poland (signature SKE 01-30/2021) in accordance with the latest revision and standards of the Helsinki Declaration. All participants signed a written consent form prior to joining the project.

### 2.2. Methods

**Figure 2:**
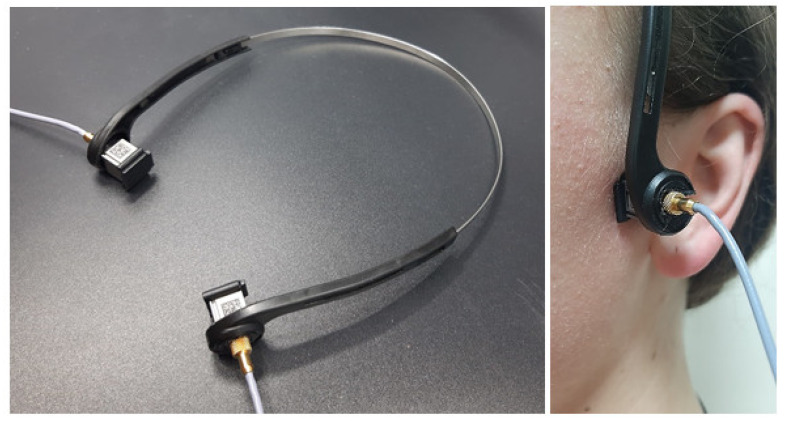
Sensors and their placement on the subject’s joints.

The examination was performed with the subjects in a seated position. The VAG sensors were mounted on a headband placed over the subject’s TMJs (both right and left at the same time) and fixed with a double-sided adhesive tape to avoid any excessive motion during the jaw movements (Figure 2). The location of the joints was determined by palpation performed by a single TMD specialist.

Prior to examination, the subjects were instructed on how to perform the procedure during the recordings. Each recording session lasted for 20 s, and comprised 10 cycles of maximal unassisted opening and closing of the jaw guided by a metronome set at 80 beats per minute. The recording sessions were performed twice, with a 5-min break between them with the removal and replacement of the device. Sensor placement and all recordings were performed by the same examiner.

The VAG signals were collected with accelerators (model 4513B-002) and a multichannel Nexus conditioning amplifier (Brüel & Kjær Sound and Vibration Measurement A/S, Denmark). The data were recorded at a frequency of 5 kHz, signal amplitude of 2 mV, and recording time of 20 s. The mentioned parameters were chosen on the basis of the pilot study. The TiePie Multi Channel (version 1.44.11000/0.9.15.3, www.tiepie.com) was used for the recordings.

### 2.3. Statistical Analysis

To obtain the intraclass correlation coefficient (ICC), the two-way mixed-effects, absolute-agreement, multiple-measurement model was chosen [40], i.e., ICC(A,1), as defined in [41]. Confidence intervals (lower: $CI_{L}$ and upper: $CI_{U}$) were calculated in accordance with [42]. Specific equations used to obtain both the ICC and the confidence intervals are included in Appendix A.

The ICC was calculated for three groups: separate study group (symptomatic), separate control group (asymptomatic), and the two groups combined. In each group, the ICC was determined for seven VAG features. According to [43], the sample size used in this study (94 subjects) was enough to estimate an ICC greater than around 0.7. The control and the study group comprised 47 subjects each, which, according to [43], was enough to estimate ICC above around 0.8. Comparing the ICC values calculated for the separate groups to those for the combined group allowed for us to draw additional conclusions about the differentiation capabilities of the specific features. When the ICC for the combined group was lower than the ICC of the separate groups, we could assume that the feature could be used to distinguish between the separate classes, since a lower ICC value indicates greater variability between subjects.

### 2.4. Vag Signal Features

Seven VAG features were calculated in accordance with the previous research mentioned earlier. All features were defined in the frequency domain on the spectrum obtained via fast Fourier transform [31]. Those features were:

1. Total integral (TI): area under the spectrum curve.
2. Integral below 300 Hz (IB3): area under the spectrum up to the 300 Hz mark.
3. Integral above 300 Hz (IA3): area under the spectrum above the 300 Hz mark.
4. Ratio of integral below and above 300 Hz (IBAR): area under the spectrum up to the 300 Hz mark divided by the area under the spectrum curve above the 300 Hz mark.
5. Peak amplitude (PA): value of the highest amplitude of the spectrum.
6. Peak frequency (PF): value of the frequency at which peak amplitude occurred.
7. Median frequency (MF): value of the frequency at which areas under the curves above and below it are equal.

### 2.5. Classification

To test the classification capabilities of the studied features, we constructed a simple k-nearest neighbors (KNN) classifier and compared it to the JVA decision tree classifier [33].

The KNN classifier [44] was defined for 10 neighbors and Euclidean distance. To improve classification, features were standardized, i.e., rescaled to have a mean value of 0 and standard deviation of 1 [45]. Classification metrics were obtained for 10-fold cross-validation [46].

Previous studies reported the VAG spectrum to be of 0.1 Hz resolution in the 0 to 1000 Hz range [31] or 0 to 500 Hz with a resolution of 1 Hz [47]. In our setup, the VAG signal was obtained with 5 kHz sampling frequency and lasted for 20 s, resulting in frequencies up to 2500 Hz with a resolution of 0.05 Hz.

Therefore, three types of features were used as the JVA decision tree classifier:

1. *Raw* features, i.e., features obtained for the 0–2500 Hz range with a resolution of 0.05 Hz.
2. *Norm1* features, i.e., features obtained for the spectral curve up to 1 kHz and resampled to a resolution of 0.1 Hz [31].
3. *Norm2* features, i.e., features obtained for the spectral curve up to 500 Hz, resampled to a resolution of 1 Hz [47].

Each resampling was performed using simple linear interpolation. Additionally, the ICC was computed for each feature defined on those two resampled spectra, for which the results are included in Appendix C.

Classification results were, therefore, obtained for 8 setups: the JVA tree classifier with *raw*, *norm1*, and *norm2* features, and the KNN classifier for *raw* features. Each classification was performed for two signal groups: the first and second measurements.

## 3. Results

Intraclass correlation coefficients of the *raw* features with the corresponding 95% confidence intervals are included in Table 1. The values of the ICC for the computed features using normalized spectra are included in Appendix C (Table A2, Table A3 and Table A4).

**Figure 3:**
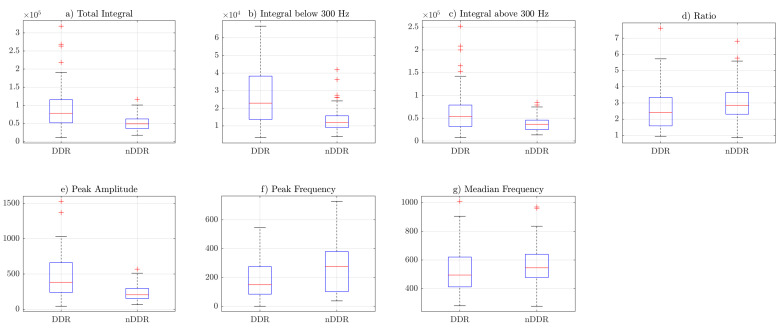
Box plots of *raw* features: (**a**) TI feature, (**b**) IB3 feature, (**c**) IA3 feature, (**d**) IBAR feature, (**e**) PA feature, (**f**) PF feature, (**g**) MF feature.

**Figure 4:**
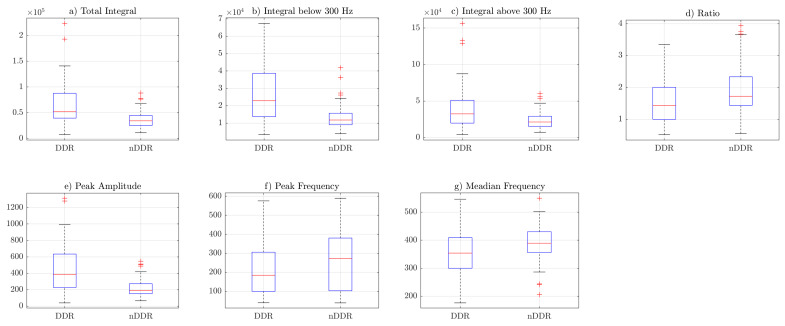
Boxplots of the *norm1* features: (**a**) TI feature, (**b**) IB3 feature, (**c**) IA3 feature, (**d**) IBAR feature, (**e**) PA feature, (**f**) PF feature, (**g**) MF feature.

**Figure 5:**
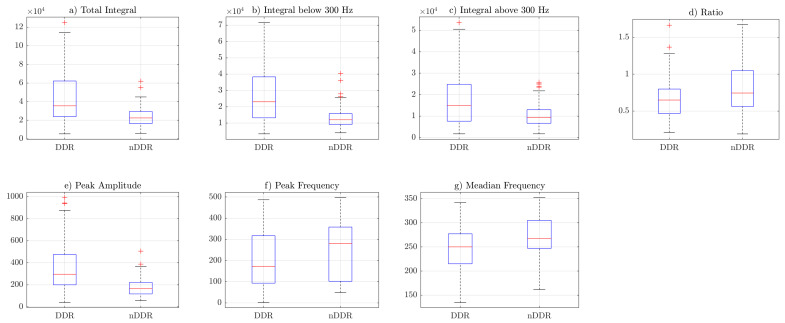
Box plots of *norm2* features: (**a**) TI feature, (**b**) IB3 feature, (**c**) IA3 feature, (**d**) IBAR feature, (**e**) PA feature, (**f**) PF feature, (**g**) MF feature.

**Figure A1:**
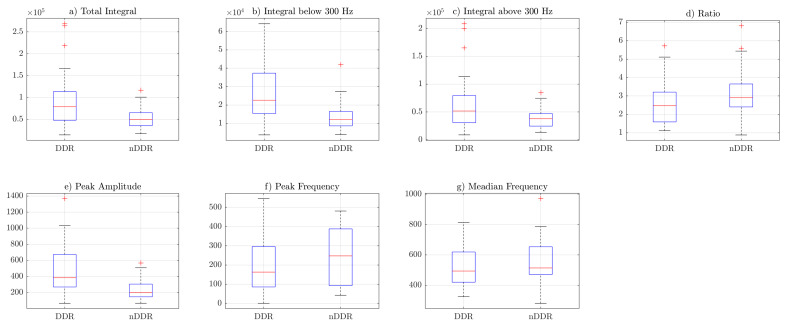
Box plots of *raw* features obtained for the first measurement: (**a**) TI feature, (**b**) IB3 feature, (**c**) IA3 feature, (**d**) IBAR feature, (**e**) PA feature, (**f**) PF feature, (**g**) MF feature.

**Figure A2:**
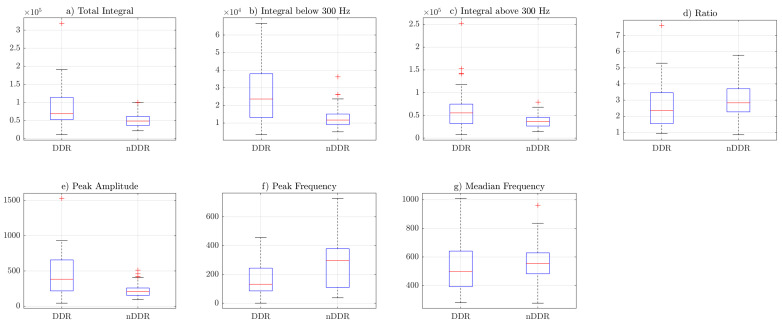
Box plots of *raw* features obtained for the second measurement: (**a**) TI feature, (**b**) IB3 feature, (**c**) IA3 feature, (**d**) IBAR feature, (**e**) PA feature, (**f**) PF feature, (**g**) MF feature.

**Figure A3:**
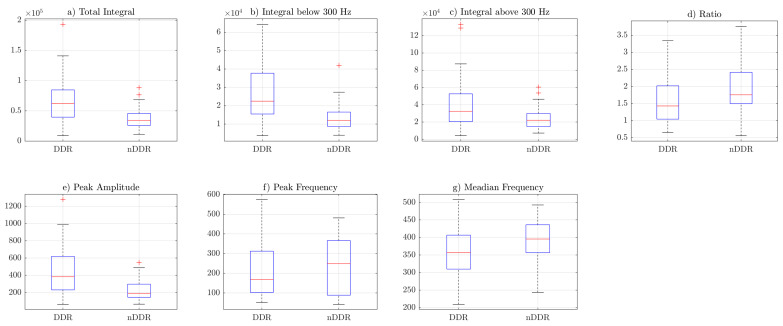
Box plots of *norm1* features obtained for the first measurement: (**a**) TI feature, (**b**) IB3 feature, (**c**) IA3 feature, (**d**) IBAR feature, (**e**) PA feature, (**f**) PF feature, (**g**) MF feature.

**Figure A4:**
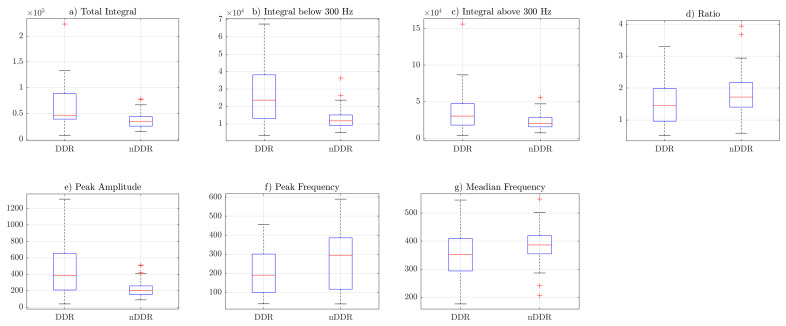
Box plots of *norm1* features obtained for the second measurement: (**a**) TI feature, (**b**) IB3 feature, (**c**) IA3 feature, (**d**) IBAR feature, (**e**) PA feature, (**f**) PF feature, (**g**) MF feature.

**Figure A5:**
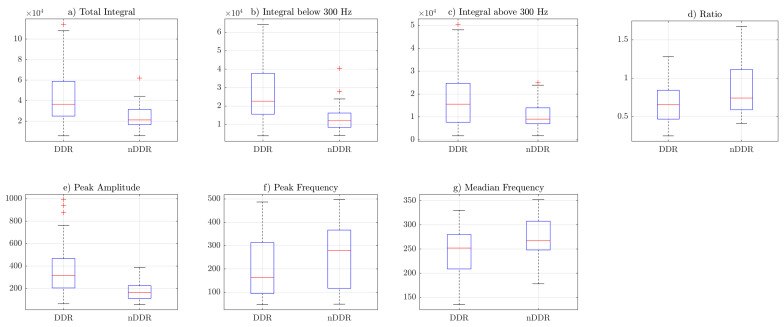
Box plots of *norm2* features obtained for the first measurement: (**a**) TI feature, (**b**) IB3 feature, (**c**) IA3 feature, (**d**) IBAR feature, (**e**) PA feature, (**f**) PF feature, (**g**) MF feature.

**Figure A6:**
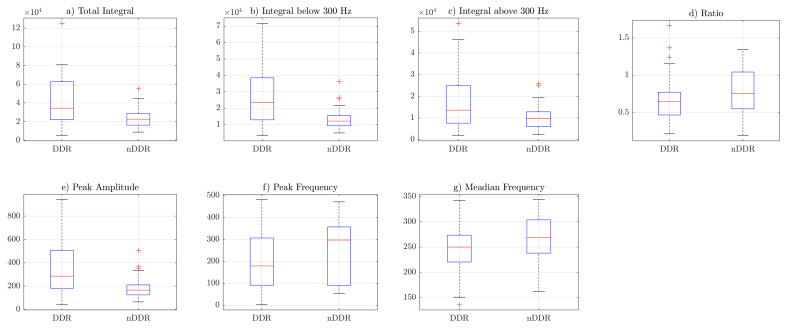
Box plots of *norm2* features obtained for the second measurement: (**a**) TI feature, (**b**) IB3 feature, (**c**) IA3 feature, (**d**) IBAR feature, (**e**) PA feature, (**f**) PF feature, (**g**) MF feature.

Figure 3, Figure 4 and Figure 5 include the box plots of *raw*, *norm1* and *norm2* features, respectively. The blue box represents the interquartile range of samples inside the class, the red line inside the box indicates the median, and the whiskers represent the rest of the values that were not considered to be outliers. Outliers are denoted by red crosses. The box plots were generated including the values of the features from both measurements. Corresponding figures for the first and the second measurements are separately included in Appendix D (Figure A1, Figure A2, Figure A3, Figure A4, Figure A5 and Figure A6).

**Figure A7:**
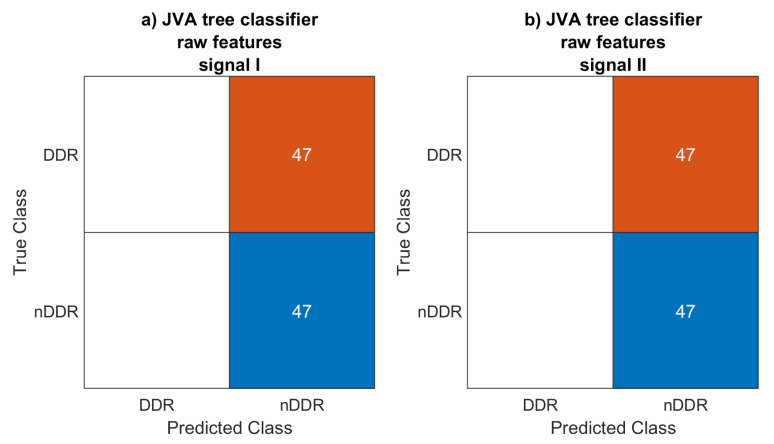
Confusion matrices for *raw* features used in the JVA decision tree classifier for the (**a**) first and (**b**) second signals.

**Figure A8:**
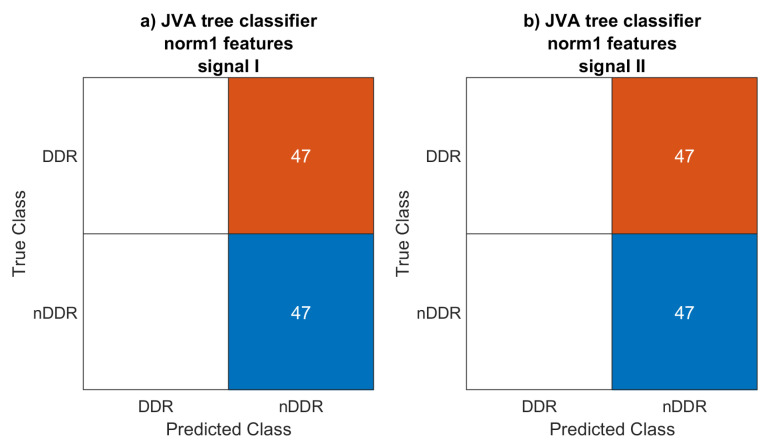
Confusion matrices for *norm1* features used in the JVA decision tree classifier for the (**a**) first and (**b**) second signals.

**Figure A9:**
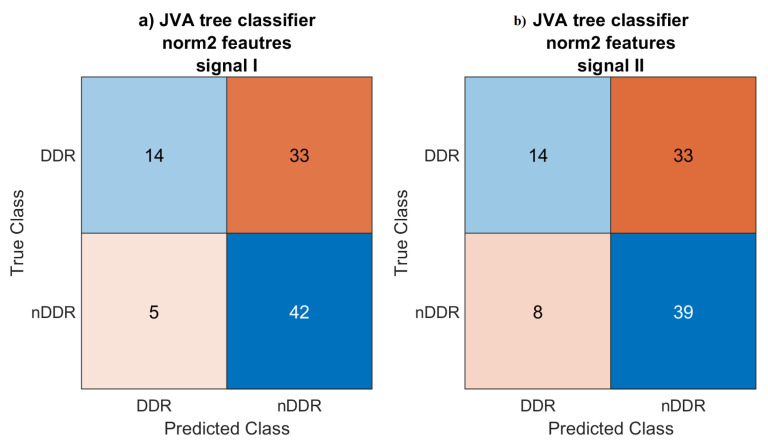
Confusion matrices for *norm2* features used in the JVA decision tree classifier for the (**a**) first and (**b**) second signals.

**Figure A10:**
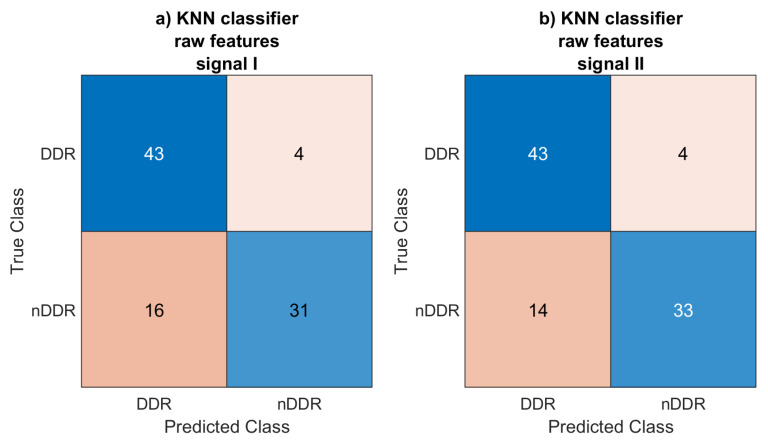
Confusion matrices for *raw* features used in the KNN classifier for the (**a**) first and (**b**) second signals.

A comparison of the classification metrics (true-positive and true-negative rates, and accuracy) between different classifiers is shown in Table 2. The confusion matrices of each classifier are3 included in Appendix E (Figure A7, Figure A8, Figure A9 and Figure A10).

## 4. Discussion

### 4.1. ICC

According to Koo and Li [40], ICC values can indicate poor, moderate, good, and excellent reliability when they fall within the ranges of 0.0–0.5, 0.5–0.75, 0.75–0.9, and 0.9–1.0, respectively. For the combined groups, ICC indicated excellent reliability for the integral below 300 Hz feature. The total integral, integral above 300 Hz, and peak amplitude features had good reliability. Moderate reliability was determined for the IBAR and median frequency features. Only the peak amplitude had an ICC value below 0.5, indicating poor reliability. However, according to [43], for the sample size of 94 participants used in this study, only ICC values of around 0.7 and above were reasonably interpretable.

The values of the ICC calculated for the separate control group were generally much lower, with only two features, i.e., total integral and the integral above 300 Hz, indicating good reliability. This means that the variability of the features’ values between participants was just a little greater than that between the measurements of the same participant. This can indicate that VAG signals for participants in the control group were similar in terms of mentioned features. This was to be expected to some extent, since the features were defined to differentiate between symptomatic and asymptomatic joints in the first place. The values of the study group were generally significantly greater than those of the control group. This means that the variability between participants was much greater than that between measurements of the same joint. Again, however, the specific values of the ICC determined for the control and study groups alone are to be taken with a grain of salt. The reliable interpretation of ICC values with a sample of 47 subjects is possible for values above around 0.8 [43]. Additional tables regarding the power analysis of the ICC calculation [48] are included in Appendix B.

### 4.2. Differences in Features between Groups

Box plots from Figure 3, Figure 4 and Figure 5 indicate that the values of the total integral, integral below 300 Hz, integral above 300 Hz, and peak amplitude features were higher for the symptomatic group. This suggests that, in general, symptomatic joints generate signals of higher energy. The ratio (IBAR) feature indicates that, for most signals in both groups, for the *raw* and *norm1* features, the integral above 300 Hz was lower than the integral below 300 Hz. This difference was more profound in the asymptomatic group. Figure 5 shows that, for *norm2* features (i.e., spectrum resampled to 1 Hz resolution up to 500 Hz), the ratio was also higher is control group. However, because of spectral normalization, the values of the IBAR feature were generally lower than 1, i.e., the integral above 300 Hz was higher than the integral below 300 Hz. The peak frequency and median frequency features indicate that the spectrum tended to lower values in the symptomatic group.

### 4.3. Classification

The JVA decision tree classifier did not work with the *raw* and *norm1* features, classifying each signal as generated by an asymptomatic joint. A couple of signals for which the features were computed using the *norm2*-normalized spectrum were classified as symptomatic. For this classification setup, accuracy was 0.596 and 0.564 for the first and the second measurements, respectively. Low accuracy is to be expected, however, since the JVA decision tree [33] was defined for a completely different signal acquisition setup.

The KNN classifier achieved 0.787 and 0.809 accuracy scores for the first and the second signals, respectively. This may be considered to be rather low. However, only features known from the literature using different setups were considered.

Features describing the vibroarthrogram in the same measurement setup but a different joint [6,49] could prove to be much more informative. This will be carried out in future studies. Frequency range maps [49] could also be obtained using independent feature evaluation coefficients of the classification algorithm [50].

### 4.4. Comparison to Previous Research

Designations can be found in the literature on the use of JVA features of vibroacoustic signals that best characterize individual TMJ pathologies. The research results indicate that the characteristics of vibroacoustic signals that best describe disk displacement with reduction (IIa according to the (R)DC/TMD) are: (1) high peak amplitude values [33], (2) total integral values above 80 Pa (even reaching 1000 Pa in sharp displacements) [33], (3) and high integral parameter below 300 Hz values [33,51].

In the current study, these parameters were characterized by good (PA and TI) and excellent (IB3) repeatability between the two measurements, which suggests that they would be valuable predictors in differentiating study-group joint clicks from those of the control group. Further analysis of the results confirmed this assumption, because all of them (PA, TI, and IB3) significantly differed in both groups, where the presence (or lack) of joint clicks was the main criterion for qualifying participants to the study and control groups. PA and IB300 features showed the highest differences between the groups among all analyzed features of the VAG signals. All “integral” features were higher for the symptomatic group, further confirming the previous findings.

In connection with the postulation outlined by the authors of the above publications to conduct further research on this problem, work has emerged over the years showing that vibroacoustic signals registered in the temporomandibular joint are repeatable [4,5,31]. The same results were presented by the authors using a piezoelectric accelerometer while examining other peripheral joints of the knee or ankle [6,28,37].

In conclusion, the results of this research are in line with previous reports on the proposed parameters of vibroacoustic signals best illustrating joint clicks.

The previous literature of the acoustic emission analysis of different joints (such as the knee joint) [52,53,54] is quite informative, especially in the context of healthy/osteoarthritis distinction (classification accuracies of over 90%). Therefore, VAG analysis in the TMJ context requires much more further research, especially in the area of feature extraction.

### 4.5. Further Research

Currently, there are only a few defined features of the vibration signals of the TMJ joint. However, by providing evidence for the repeatability of signals in the proposed measurement chain in this study, an entirely new field of research has opened up.

Future research could benefit from focusing on the VAG features used to diagnose other joints, such as the knee. This would allow for a more accurate diagnosis of the TMJ. Exemplary features could include previously used measures in knee-joint VAG analysis. Those include time-domain features, such as rectified average value, root mean square value, and shape factors [55,56], and frequency-domain features, such as spectral entropy [57]. Additional preprocessing such as (ensemble) empirical mode decomposition [58,59] could also be studied in the context of TMJ.

Additionally, since the measurement chain used in this study was accurate for both the TMJ and knee joint [6], it would be of great value to study vibroarthrographic signals in the context of other joints.

### 4.6. Conclusions

Most of the investigated features showed moderate-to-excellent reliability, indicating a high repeatability of VAG signals, thus allowing for diagnostic usage for further research.

Temporomandibular joint sound diagnostics using the KNN classifier showed higher accuracy scores compared with those of the JVA decision tree, but the results could still be considered to be rather low.

## Figures and Tables

**Table 1:** Intraclass correlation coefficients (ICC) for each feature with corresponding 95% confidence intervals (CIs).

		Study Group				Control Group				Combined		
Feature		LCI	ICC	UCI		LCI	ICC	UCI		LCI	ICC	UCI
TI		0.826	0.871	0.905		0.685	0.761	0.821		0.779	0.881	0.937
IB3		0.878	0.910	0.934		0.581	0.677	0.755		0.817	0.902	0.949
IA3		0.795	0.847	0.887		0.672	0.752	0.814		0.736	0.856	0.924
IBAR		0.707	0.778	0.834		0.248	0.392	0.519		0.356	0.610	0.780
PA		0.782	0.837	0.879		0.436	0.557	0.657		0.723	0.848	0.919
PF		0.024	0.183	0.332		0.291	0.431	0.553		0.030	0.352	0.606
MF		0.669	0.748	0.811		0.268	0.410	0.534		0.356	0.609	0.779

**Table 2:** Classification comparison for different normalizations. TPR, true-positive rate; TNR, true-negative rate; ACC, accuracy.

Classifier	TPR	TNR	ACC
JVA—*raw* features, the first signal	0.000	1.000	0.500
JVA—*raw* features, the second signal	0.000	1.000	0.500
JVA—*norm1* features, the first signal	0.000	1.000	0.500
JVA—*norm1* features, the second signal	0.000	1.000	0.500
JVA—*norm2* features, the first signal	0.298	0.894	0.596
JVA—*norm2* features, the second signal	0.298	0.830	0.564
KNN—*raw* features, the first signal	0.915	0.660	0.787
KNN—*raw* features, the second signal	0.915	0.702	0.809

## Data Availability

Data available on request due to privacy restrictions. The data presented in this study are available on request from the corresponding author. The data are not publicly available due to privacy restrictions.

## Abbreviations

The following abbreviations are used in this manuscript:

DC/TMD	Diagnostic Criteria for Temporomandibular Disorders
IA3	Integral above 300 Hz
IB3	Integral below 300 Hz
IBAR	Integral below 300 Hz to integral above 300 Hz ratio
ICC	Intraclass correlation coefficient
JVA	Joint vibration analysis
MF	Median frequency
MSBM	Mean square between measurements
MSBS	Mean square between subjects
MSE	Mean square error
PA	Peak amplitude
PF	Peak frequency
RDC/TMD	Research Diagnostic Criteria for Temporomandibular Disorders
TI	Total integral
TMD	Temporomandibular disorders
TMJ	Temporomandibular joints
VAG	Vibroarthrography

## Appendix A. Equations Used to Calculate the ICC and It’s Confidence Intervals

To obtain the intraclass correlation coefficient (icc), the two-way mixed-effects, absolute-agreement multiple-measurements model was chosen [40], i.e., ICC(A,1), as defined in [41]:

(A1) $$ICC=\frac{MSBS-MSE}{MSBS+(k-1)\cdot MSE+(k/n)\cdot (MSBM-MSE)},\;\mathrm{where}:$$

MSBS is the value of the mean square between subjects, MSE is the mean square error, MSBM is the mean square between measurements, *k* is the number of measurements for each subject, and *n* is the number of subjects. Confidence intervals (lower: $CI_{L}$ and upper: $CI_{U}$) were calculated in accordance with [42]:

(A2) $$CI_{L}=\frac{n\cdot (MSBS-F_{*}MSE)}{F_{*}\left[k\cdot MSBM+\left(kn-k-n\right)\cdot MSE\right]+n\cdot MSBS}$$

(A3) $$CI_{U}=\frac{n\cdot (F^{*}MSBS-MSE)}{k\cdot MSBM+\left(kn-k-n\right)\cdot MSE+n\cdot F^{*}\cdot MSBS}$$

where $F_{*}$ and $F^{*}$ denote 97.5-th percentiles of the *F* distribution with n−1 degrees of freedom (DOF) in the numerator and ν DOF in the denominator for $F_{*}$, and inversely for $F^{*}$.

(A4) $$\nu =\frac{{\left(a\cdot MSBM+B\cdot MSE\right)}^{2}}{\frac{{\left(a\cdot MSBM\right)}^{2}}{k-1}+\frac{{\left(b\cdot MSE\right)}^{2}}{\left(n-1)\cdot (k-1\right)}}$$

(A5) $$a=\frac{k\cdot (ICC)}{n\cdot (1-ICC)}$$

(A6) $$b=1+\frac{k\cdot ICC\cdot (n-1)}{n\cdot (1-ICC)}$$

## Appendix B. Post Hoc Power Analysis

Post hoc power analysis was conducted for the ICC values from Table 1 according to [48]. Power values were obtained for an alpha level of 0.05, and the null hypothesis of ICC was equal to 0.0 and 0.5. Results are included in Table A1.

**Table A1:** Post hoc power analysis of the ICC for each feature in Table 1. The columns of power (0.0) and power (0.5) correspond to power values obtained for the null hypothesis of ICC equal to 0.0 and 0.5, respectively.

		Study Group				Control Group				Combined		
Feature		ICC	Power (0.0)	Power (0.5)		ICC	Power (0.0)	Power (0.5)		ICC	Power (0.0)	Power (0.5)
TI		0.871	1.000	1.000		0.761	1.000	0.862		0.881	1.000	1.000
IB3		0.910	1.000	1.000		0.677	1.000	0.460		0.902	1.000	1.000
IA3		0.847	1.000	0.997		0.752	1.000	0.828		0.856	1.000	1.000
IBAR		0.778	1.000	0.915		0.392	0.802	0.000		0.610	1.000	0.337
PA		0.837	1.000	0.994		0.557	0.990	0.077		0.848	1.000	1.000
PF		0.183	0.241	0.000		0.431	0.879	0.000		0.352	0.944	0.000
MF		0.748	1.000	0.811		0.410	0.840	0.000		0.609	1.000	0.331

## Appendix C. Expansion of ICC Analysis

**Table A2:** Intraclass correlation coefficients (ICC) for each feature computed on *norm1*-normalized spectrum with corresponding 95% confidence intervals (CI).

		Study Group				Control Group				Combined		
Feature		LCI	ICC	UCI		LCI	ICC	UCI		LCI	ICC	UCI
TI		0.676	0.902	0.928		0.676	0.755	0.949		0.817	0.902	0.949
IB3		0.579	0.907	0.932		0.579	0.676	0.947		0.813	0.900	0.947
IA3		0.665	0.885	0.915		0.665	0.746	0.937		0.779	0.881	0.937
IBAR		0.299	0.785	0.839		0.299	0.438	0.782		0.362	0.614	0.782
PA		0.465	0.853	0.891		0.465	0.581	0.926		0.743	0.860	0.926
PF		0.163	0.434	0.555		0.163	0.314	0.624		0.059	0.377	0.624
MF		0.001	0.843	0.884		0.001	0.161	0.769		0.334	0.594	0.769

**Table A3:** Intraclass correlation coefficients (ICC) for each feature computed on *norm2*-normalized spectrum with corresponding 95% confidence intervals (CI).

		Study Group				Control Group				Combined		
Feature		LCI	ICC	UCI		LCI	ICC	UCI		LCI	ICC	UCI
TI		0.877	0.909	0.934		0.822	0.719	0.788		0.822	0.905	0.950
IB3		0.860	0.896	0.924		0.800	0.658	0.740		0.800	0.893	0.944
IA3		0.860	0.896	0.924		0.777	0.704	0.777		0.777	0.880	0.937
IBAR		0.636	0.722	0.790		0.373	0.507	0.616		0.373	0.621	0.786
PA		0.691	0.766	0.825		0.629	0.537	0.641		0.629	0.791	0.887
PF		0.004	0.164	0.316		0.000	0.378	0.507		0.000	0.286	0.558
MF		0.705	0.777	0.833		0.506	0.579	0.676		0.506	0.712	0.842

**Table A4:** Best normalization for intraclass correlation coefficients (ICC) based on Table 1, Table A2, and Table A3.

		Study Group				Control Group				Combined		
Feature		LCI	ICC	UCI		LCI	ICC	UCI		LCI	ICC	UCI
TI		*norm2*	*norm2*	*norm2*		*norm2*	*raw*	*norm1*		*norm2*	*norm2*	*norm2*
IB3		*raw*	*raw*	*raw*		*norm2*	*raw*	*norm1*		*raw*	*raw*	*raw*
IA3		*norm2*	*norm2*	*norm2*		*norm2*	*raw*	*norm1*		*norm1*	*norm1*	*norm1*
IBAR		*raw*	*norm1*	*norm1*		*norm2*	*norm2*	*norm1*		*norm2*	*norm2*	*norm2*
PA		*raw*	*norm1*	*norm1*		*norm2*	*norm1*	*norm1*		*norm1*	*norm1*	*norm1*
PF		*norm1*	*norm1*	*norm1*		*raw*	*raw*	*norm1*		*norm1*	*norm1*	*norm1*
MF		*norm2*	*norm1*	*norm1*		*norm2*	*norm2*	*norm1*		*norm2*	*norm2*	*norm2*

## Appendix D. Boxplots for Separate Measurements

Figure A1, Figure A2, Figure A3, Figure A4, Figure A5 and Figure A6 include box plots of each feature for the first and the second measurements, respectively. Box plots were very similar between the measurements. Therefore, the interpretation of the box plots obtained for the separate measurements was the same as that of the combined measurements (box plots in Figure 3, Figure 4 and Figure 5).
